# Survival of critically ill people with COVID-19 and acute kidney injury undergoing hemodialysis in public and private hospitals in Joinville: a cohort study, 2020-2021

**DOI:** 10.1590/S2237-96222025v34e20240025.en

**Published:** 2025-04-07

**Authors:** Elviani Basso Moura, Bruna de Albuquerque Catelano, Fernanda Perito de Aguiar, Helbert do Nascimento Lima, Paulo Henrique Condeixa de França

**Affiliations:** 1 Universidade da Região de Joinville, Programa de Pós-Graduação em Saúde e Meio Ambiente, Joinville, SC, Brazil

**Keywords:** COVID-19, Acute Kidney Injury, Brazilian National Health System, Supplementary Health, Survival Rate, COVID-19, Insuficiencia Renal Aguda, Sistema Único de Salud, Salud Suplementaria, Tasa de Supervivencia.

## Abstract

**Objective:**

To compare the 90-day survival of critically ill people with COVID-19 and acute kidney injury in intensive care units (ICU) of public and private hospitals.

**Methods:**

This was a retrospective cohort study of critically ill people with COVID-19 and acute kidney injury undergoing hemodialysis in Joinville, Santa Catarina state.

**Results:**

The 90-day survival rate in public ICU was 15.7% (95%CI 8.4; 25.1), while in private ICU it was 37.7% (95%CI 24.9; 50.5%). In the multivariate analysis adjusted for sociodemographic variables (Hazard ratio (HR) 2.01; 95%CI 1.31; 3.08) and comorbidities (HR 2.09; 95%CI 1.30; 3.37), admission to a public ICU was associated with a higher risk of death. After including severity of illness, admission to a public ICU was not associated with an increased risk of death compared to private ICU (HR 0.79; 95%CI 0.45; 1.42).

**Conclusion:**

Measures implemented during the COVID-19 pandemic may have reduced inequalities between health systems for critically ill patients with acute kidney injury.

## Resumen

**Objetivo:**

Comparar la supervivencia a 90 días de pacientes críticos con COVID-19 e insuficiencia renal aguda en unidades de cuidados intensivos (UCI) de hospitales públicos y privados.

**Métodos:**

Se trata de un estudio de cohorte histórica de pacientes críticos en Joinville con COVID-19 e insuficiencia renal aguda sometidos a hemodiálisis.

**Resultados:**

La supervivencia a 90 días en las UCI públicas fue del 15,7% (IC95% 8,4; 25,1), mientras que en las UCI privadas fue del 37,7% (IC95% 24,9; 50,5). En el análisis multivariable ajustado por variables sociodemográficas (Hazard ration (HR) 2,01; IC95% 1,31; 3,08) y comorbilidades (HR 2,09; IC95% 1,30; 3,37), la hospitalización en UCI pública se asoció con un mayor riesgo de muerte. Sin embargo, tras incluir la gravedad en el modelo, la hospitalización en UCI pública no mostró asociación con un mayor riesgo de muerte en comparación con las UCI privadas (HR 0,79; IC95% 0,45; 1,42).

**Conclusión:**

Las medidas adoptadas durante la pandemia de COVID-19 pueden haber reducido las desigualdades entre los sistemas de salud para los pacientes críticos con insuficiencia renal aguda.

## Introduction

The incidence of acute kidney injury (AKI) is high among people with COVID-19 receiving care in intensive care units (ICU). AKI has been reported in 31.5% of people with COVID-19 who were hospitalized, reaching up to 77.3% of critically ill people 9 ^1^ ^,^ ^2^. Renal impairment is associated with increased morbidity and mortality, longer hospital stays and the need for dialysis in people with COVID-19 who were hospitalized ^3^ ^,^ ^4^. The likelihood of death in people with COVID-19 and AKI is higher than in those without AKI ^5^.

In a retrospective study involving people with COVID-19 and AKI in ICU, IKA was identified in 75% of cases, with half of people requiring dialysis and a 60-day mortality rate of up to 67% ^6^. Factors such as advanced age, male sex, diabetes, hypertension, cardiovascular disease and respiratory failure requiring mechanical ventilation were associated with a higher incidence of AKI in people with severe COVID-19 ^1^ ^,^ ^5^ ^,^ ^7^.

The fight against the COVID-19 pandemic in Brazil was marked by disparities in healthcare provision and access to hospital beds ^8^. Severe cases of the disease had the greatest impact on both public and private healthcare systems, with a rapid increase in the demand for hospitalizations, especially in ICU ^9^. Significant efforts from healthcare managers were required to address COVID-19 ^11^, given that 80% of the population relied exclusively on the Brazilian National Health System (Sistema Único de Saúde - SUS) ^10^.

Expanding the SUS has been a constant challenge in meeting the healthcare needs of the Brazilian population, with a dramatic increase in health service coverage over just three decades ^12^. Significant disparities in access opportunities and availability of ICU beds within the SUS were observed (1.78 total beds and 0.25 ICU beds per 1,000 inhabitants), reflecting persistent healthcare inequities ^13^. SUS is the primary provider of access to healthcare services to the population. The system also serves users of private health insurance plans when specialized or high-complexity care is not fully covered by their insurance ^10^.

Mortality data from critically ill people with COVID-19 and AKI highlighted weaknesses in the SUS, being 65.4% versus 23.8% in the private health service ^14^. Little is known about the impact of COVID-19-associated AKI on critically ill people receiving care within the SUS when compared to those receiving care in the private health system.

Challenges have emerged in the management of health services in the face of the outbreak of the COVID-19 pandemic, which required the reorganization of hospitals and the care provided to critically ill people. This study aimed to compare the 90-day survival of critically ill people with COVID-19 and AKI requiring hemodialysis and receiving care in the public and private health services in Joinville, the third most populous city in Southern Brazil. The study investigated whether the type of care, public or private, influenced mortality in these patients.

## Methods

### Design

This is a retrospective cohort study based on electronic medical records. All adult patients admitted to ICU in three hospitals in Joinville, Santa Catarina state, with a diagnosis of COVID-19 and requiring acute hemodialysis due to AKI, between March 2020 and February 2021, were included. COVID-19 infection was confirmed using molecular testing or rapid antigen testing. 

### Participants

Three hospitals participated in the study. Two were private (Hospital Dona Helena and Centro Hospitalar Unimed) and one was exclusively public (Hospital Municipal São José). All ICU were staffed by multidisciplinary teams, consisting of intensivists, nutritionists, nurses and physiotherapists. These hospitals were affiliated with medical residency programs. During the study period, all hospitals expanded their ICU bed capacity to meet the increased demand for COVID-19 cases. Hospital Municipal São José had 40 ICU beds; Hospital Dona Helena had 18; and Centro Hospitalar Unimed had 20 ICU beds.

### Variables

Based on data contained in the medical records, demographic information (sex and age) and current and previous comorbidities, such as hypertension, diabetes, respiratory diseases (chronic obstructive pulmonary disease or asthma), cerebrovascular and cardiovascular diseases (stroke, acute myocardial infarction, myocardial revascularization or coronary angioplasty) and cancer were collected. Data on the Simplified Acute Physiology Score III (SAPS-3), related to the severity and mortality prediction in the ICU, information on ventilatory support requirements and duration, presence of acute kidney injury requiring hemodialysis, length of ICU stay, and in-hospital mortality.

AKI was diagnosed by a nephrologist in each participating hospital, and the indication for hemodialysis was determined based on clinical (hypervolemia) and laboratory parameters (metabolic acidosis, azotemia or hyperkalemia). All patients with an indication for dialysis underwent intermittent hemodialysis using standard bicarbonate bath and temporary vascular access.

### Statistical methods

Categorical variables were presented based on their frequency and percentage. Quantitative variables were presented by their mean and standard deviation or median and interquartile range, according to their distribution. The chi- square test or Fisher’s exact test were used to compare categorical variables. The Mann Whitney test, following confirmation of non-normal distribution through the Kolmogorov -Smirnov test, was used to compare the means of quantitative variables according to the main exposure variable, type of hospital (public or private), as well as between survivors or non-survivors during 90 days of hospitalization. Follow-up time was defined from the person’s ICU admission to in-hospital death within 90 days. Survivors discharged from the hospital before 90 days were censored after the time of discharge.

The survival curve analysis according to the type of hospital was performed using the Kaplan-Meier method, with the difference assessed by means of Log-Rank test. The HR for the type of hospital was calculated in crude and adjusted forms for main confounding variables using bivariate Cox regression. Confounding variables were defined as those known to be associated with the main exposure or representing a risk factor for the outcome. Variables that modified the effect between the main exposure (type of hospital) and the outcome (90-day mortality) by 5% or more in the bivariate analysis were included in the multivariate model. To better visualize the change in the effect between the main variable of interest in relation to the outcome, variables considered in the multivariate model were included in blocks, starting with sociodemographic variables, followed by comorbidities and complications/severity of people in the ICU. All calculations were performed using the Stata/IC 15.1 software.

## Results

During the study period, 123 (22.9%) of the 538 people admitted to ICU in public and private hospitals with COVID-19 developed AKI requiring acute hemodialysis. Seventy people (56.9%) were admitted to a public hospital, and 53 (49.1%) to private hospitals.

The median age of the total sample analyzed was 63 years, with 79 (64.2%) men. The most common comorbidities were hypertension (n=72; 58.5%) and diabetes (n=44; 35.8%). Mechanical ventilation was required in 118 (95.9%) cases, the median SAPS-3 was 67 (interquartile range 52/76) and 92 (74.8%) people died within 90 days. Compared to people admitted to a private hospital, people admitted to a public hospital showed a lower prevalence of chronic obstructive pulmonary disease or asthma (5.7% vs. 22.6%; p-value 0.007), a higher need for mechanical ventilation (100% vs. 90.6%; p-value 0.009), shorter median ICU stay (17.5 vs. 23 days; p-value 0.022), shorter median duration of mechanical ventilation (17 vs. 21.5 days; p-value 0.031), higher median severity (73.5 vs. 52; p-value<0.001) and higher mortality rate (84.3% vs. 62.3%; p-value 0.005) (Table 1). There was no difference in age between people admitted to public hospitals (median 65 years, interquartile range 54-73) and private hospitals (median 60 years; interquartile range 47-73; p-value 0.199). ICU length of stay in private hospitals (21 days, interquartile range 12-38) was longer compared to public hospitals (17.5 days, interquartile range 10-29; p-value 0.022). Similarly, the duration of mechanical ventilation was longer in private hospital patients (21.5 days, interquartile range 14-45) compared to those in public hospitals (17 days, interquartile range 10-24; p-value 0.031). However, the severity was higher among people admitted to public hospitals (73.5, interquartile range 64-78) compared to those in private hospitals (52, interquartile range 49-64; p-value<0.001).

**Table 1:** Characteristics of people with COVID -19 and acute kidney injury requiring hemodialysis by type of hospital. Joinville, 2020-2021 (n=123)

Variables	Private (%)	Public (%)	p-value	Total (%)
Male	38 (71.7)	41 (58.6)	0.133	79 (64.2)
Hypertension	35 (66.0)	37 (52.9)	0.142	72 (58.5)
Diabetes	19 (35.8)	25 (35.7)	0.988	44 (35.8)
Respiratory disease	12 (22.6)	4 (5.7)	0.007	16 (13.0)
Smoking	8 (15.1)	11 (15.7)	0.925	19 (15.4)
Cancer	8 (15.1)	4 (5.7)	0.077	12 (9.8)
Cerebrovascular and cardiovascular diseases	6 (11.3)	3 (4.3)	0.129	9 (7.3)
Use of vasoactive drugs	32 (60.4)	48 (68.6)	0.345	80 (65.0)
Mechanical ventilation	48 (90.6)	70 (100.0)	0.009	118 (95.9)
Death	33 (62.3)	59 (84.3)	0.005	92 (74.8)

Compared to survivors, people who died showed a higher mean age (66.5 vs. 60.1 years; p-value 0.009), higher frequency of use of vasodilators (76.1% vs. 32.3%; p-value<0.001), greater need for mechanical ventilation (100% vs. 83.9%; p-value<0.001) and shorter median duration of mechanical ventilation (17 vs. 30.5 days; p-value 0.006) and shorter ICU stay (17 vs. 42 days; p-value 0.001). The median SAPS-3 was higher among people who died (70 vs. 58; p-value 0.005; Table 2). There was a difference in age between survivors (median 55 years, interquartile range 44-66) and those who died (median 70 years; interquartile range 60-77; p-value 0.009). Survivors also had longer ICU stays (42 days, interquartile range 22-89) compared to those who died (17 days, interquartile range 11-28; p-value 0.001). Similarly, survivors had longer durations of mechanical ventilation (30.5 days, interquartile range 18-52) compared to those who died (17 days, interquartile range 10-24; p-value 0.006). However, the severity was lower among survivors (58, interquartile range 49-67) compared to those who died (70, interquartile range 54-7; p-value 0.005).

**Table 2:** General characteristics of people with COVID-19 and acute kidney injury requiring hemodialysis regarding 90-day survival Joinville, 2020-2021 (n=123)

Variables	Survival (%)	Death (%)	p-value
Male	16 (51.6)	63 (68.5)	0.090
Hypertension	22 (71.0)	50 (54.3)	0.104
Diabetes	11 (35.5)	33 (35.9)	0.969
Respiratory disease	4 (12.9)	12 (13.0)	0.627
Smoking	2 (6.4)	17 (18.5)	0.089
Cancer	2 (6.4)	10 (10.9)	0.374
Cerebrovascular and cardiovascular diseases	3 (9.7)	6 (6.5)	0.405
Use of vasoactive drugs	10 (32.3)	70 (76.1)	<0.001
Mechanical ventilation	26 (83.9)	92 (100.0)	<0.001
Private hospital	20 (64.5)	33 (35.9)	0.005
Public hospital	11 (35.5)	59 (64.1)	0.005

**Figure 1:**
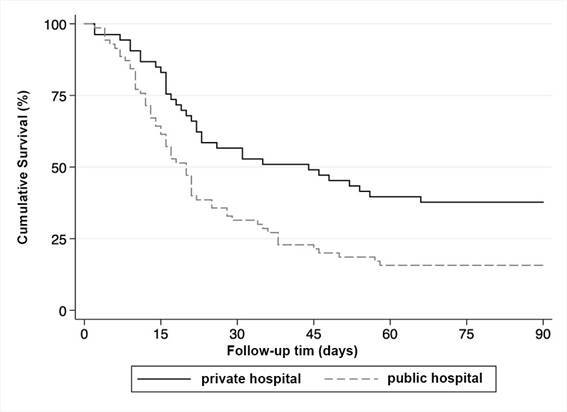
90-day survival of people with COVID-19 and dialysis-requiring acute kidney injury by type of hospital. Joiville, 2020-2021

There was a difference in survival among COVID-19 people with AKI requiring hemodialysis based on hospital type when analyzed using the Kaplan-Meier method (p-value 0.002; Figure 1). The 90-day survival of people admitted to a public hospital was 15.7% (95%CI 8.4; 25.1). The 90-day survival of people admitted to a private hospital was 37.7% (95%CI 24.9; 50.5).

People admitted to a public hospital showed a 90% higher risk of death compared to those in a private hospital (crude HR 1.93; 95%CI 1.26; 2.97). After adjusting for other variables, the effect of hospital type was modified by ≥5% when adjusted for cancer (HR 2.06; 95%CI 1.32; 3.20), chronic obstructive pulmonary disease or asthma. (HR 2.01; 95%CI 1.29; 3.05), sex (HR 1.98; 95%CI 1.29; 3.05) and age (HR 1.96; 95%CI 1.28; 3.02; Table 3).

**Table 3:** Crude and adjusted hazard ratio (HR) and crude and adjusted 95% confidence interval (95%CI) between hospital type and 90-day mortality among people with COVID-19 and acute kidney injury requiring hemodialysis.Joinville, 2020-2021 (n=123)

Variables	Crude HR (95%CI)	p-value	Adjusted HR (95%CI)	p-value
**Hospital**				
Private	1.00			
Public	1.93 (1.26; 2.97)	0.003		
Sex				
Male			1.00	
Female			1.98 (1.29; 3.05)	0.002
**Hypertension**				
No			1.00	
Yes			1.85 (1.19; 2.86)	0.006
**Diabetes**				
No			1.00	
Yes			1.93 (1.26; 2.97)	0.003
**Respiratory disease**				
No			1.00	
Yes			2.01 (1.29; 3.14)	0.002
**Cerebrovascular and cardiovascular diseases**				
No			1.00	
Yes			1.92 (1.24; 2.96)	0.003
**Cancer**				
No				
Yes			2.06 (1.32; 3.20)	0.001
**Use of vasoactive drugs**				
No			1.00	
Yes			1.68 (1.09; 2.59)	0.019
**Smoking**				
No			1.00	
Yes			1.91 (1.24; 2.94)	0.003
Age (year increase)			1.96 (1.28; 3.02)	0.002
**Duration of mechanical ventilation (days/unit** **increase)**			1.16 (0.75; 1.79)	0.501
**Severity (unit increase)**			1.35 (0.79; 2.30)	0.276

Hospitalization in a public hospital remained associated with a higher mortality risk only when adjusted for sociodemographic variables (HR 2.01; 95%CI 1.31; 3.08) and when comorbidities were included to the model (HR 2.09; 95%CI 1.30; 3.37; Table 4). When adjusted for severity, hospitalization of critically ill COVID-19 people with AKI requiring dialysis in public hospitals was not associated with a higher risk of mortality (HR 0.79; 95%CI 0.45; 1.42).

**Table 4:** Multivariate hazard ratio (HR) and confidence interval (95%CI) between type of hospital and 90-day mortality in people with COVID-19 and acute kidney injury requiring hemodialysis. Joinville, 2020-2021 (n=123)

Variables	Model 1		Model 2		Final model	
	HC (95%CI)	p-value	HC (95%CI)	p-value	HC (95%CI)	p-value
**Type of hospital (public/private)**	2.01 (1.31; 3.08)	0.001	2.09 (1.30; 3.37)	0.003	0.79 (0.45; 1.42)	0.440
**Age (year increase)**	1.02 (1.01; 1.04)	0.006	1.02 (1.00; 1.04)	0.015	1.01 (0.99; 1.03)	0.152
Sex						
male	1.00		1.00		1.00	
female	0.76 (0.48; 1.18)	0.219	0.78 (0.49; 1.24)	0.297	0.90 (0.56; 1.44)	0.654
**Diabetes**						
Yes			1.00		1.00	
No			0.99 (0.64; 1.53)	0.949	1.01 (0.64; 1.59)	0.976
**Respiratory disease**						
Yes			1.00		1.00	
No			1.19 (0.61; 2.30)	0.614	1.31 (0.64; 2.69)	0.460
**Cerebrovascular and cardiovascular diseases**						
Yes			1.00		1.00	
No			0.78 (0.33; 1.84)	0.567	0.85 (0.35; 2.02)	0.706
Cancer						
Yes			1.00		1.00	
No			1.25 (0.58; 2.67)	0.569	1.64 (0.74; 3.63)	0.223
**Use of vasoactive drugs**						
Yes					1.00	
No					1.08 (0.64; 1.84)	0.768
**Duration of mechanical** **ventilation (days/unit** **increase)**					0.96 (0.94; 0.97)	<0.001
**Severity (unit increase)**					1.03 (1.00; 1.05)	0.013

Notes: Model 1: sociodemographic variables; Model 2: comorbidity variables; Model 3: severity variables; SAPS=3: Simplified Acute Physiology Score III.

## Discussion

Significantly lower 90-day survival rates of critically ill COVID-19 people with AKI requiring dialysis admitted to a public hospital was observed. After adjusting for the severity of these people at the time of ICU admission, the type of hospital did not influence the highest 90-day mortality risk.

The presence of AKI requiring dialysis as a complicating factor for critically ill people hospitalized for COVID-19 showed a decrease throughout the pandemic, ranging from 27% in the first year to 14% in the final period of the pandemic ^15^. In this study, the overall prevalence of AKI requiring dialysis was 22.9% of people requiring ICU. The prevalence of AKI requiring dialysis in critically ill people with COVID-19 in public hospitals was 61.5% ^2^, while in private hospitals it was 17% ^14^. This variation could be partially explained by the criteria defined for dialysis indication and the severity profile of people admitted to the ICU. Among the factors associated with the AKI in people with COVID-19, the following stand out: older age, male sex, comorbidities such as hypertension, diabetes and cardiovascular disease, greater need for mechanical ventilation and higher disease severity ^16^ ^-^ ^18^. These factors were found in this study among people with dialysis-requiring AKI.

The use of mechanical ventilation in critically ill people with COVID-19 was identified as a significant risk factor for AKI ^14^ ^,^ ^19^. In a multicenter cohort conducted in public hospitals in Brazil, the use of mechanical ventilation was one of the predictors of a higher risk of mortality in people with COVID-19 ^20^. It could be seen that people admitted to public hospitals required mechanical ventilation more frequently and but had shorter length of hospital stays compared to those in the private hospitals. This shorter length of hospital stay was associated with a higher number of deaths in the public service.

The 90-day survival probability was more than twice as high for people receiving care in private hospitals when crude survival among critically ill people with COVID-19 and dialysis-requiring AKI was analyzed. Factors potentially associated with the lower survival among patients dependent on the SUS, included the use of vasodilators and mechanical ventilation. Other variables potentially contributing to lower survival in the SUS, but not assessed in this study, might include underlying social inequalities ^(10)^. and technical aspects of care ^17^ ^,^ ^21^. The time to access and type of care received prior to ICU admission could have an impact on the observed survival differences. Individual characteristics, health conditions and access to health services were determinants of survival among individuals infected with COVID-19 ^17^ ^,^ ^18^ ^,^ ^22^ ^,^ ^23^.

The risk of death at any point during the observation period, adjusted for sociodemographic variables and comorbidities, showed that public hospitals remained associated with higher mortality risk. The risk of death from COVID-19 was attributed to the high likelihood of overcrowding in public services, unequal access to health care, worse living conditions, and a higher prevalence of comorbidities ^24^. Receiving care in a public hospital in Brazil was identified as an independent factor for mortality ^25^.

The risk of death for people with COVID-19 and dialysis-requiring AKI did not differ according to the type of hospital after adjusting for sociodemographic variables and comorbidities and disease severity. Variables used to assess severity included mechanical ventilation and mortality score (SAPS-3). Mechanical ventilation was associated with the risk of mortality from COVID-19 ^26^, and the SAPS-3 was linked to higher hospital mortality in people with COVID-19 admitted to the ICU ^27^. It was noted that adjusted mortality, accounting for other factors and patient severity at ICU admission, was similar between the two hospital types in this study.

The type of health insurance did not influence access to the ICU for people with COVID-19 and chronic kidney disease already enrolled in permanent hemodialysis program ^12^. Mortality was higher among those who required public ICUs compared to private ICU ^12^. Greater severity at ICU admission and the need for mechanical ventilation among the patients who required public ICU in this study may have contributed to the fact that the mortality risk was not different from that of those who required private ICU. It was not possible to confirm that the worse situation of people admitted to the public ICU did not reflect challenges in initial care provided across other points of the public healthcare network.

Emergency expansion of healthcare structures and ICU beds may have reduced the impacts of COVID-19 on the public health system. Organizational factors, including protocol implementation and resource optimization in ICU, were considered potential measures to improve patient outcomes and reduce hospital mortality ^28^. Adult ICU beds in the SUS accounted for the majority of adult ICU beds in the country (67%) designated for COVID-19 during the study period. Santa Catarina state, where 94% of ICU beds were in the SUS, stood out ^13^.

Notable measures implemented in Joinville’s public health system included the creation of a technical task force, ICU expansion, hiring additional healthcare professionals, and purchasing equipment for severe case management ^29^. These actions may have contributed to minimizing negative outcomes for critically ill people with COVID-19 in the ICU. As shown by general COVID-19 data in Brazil, the mortality rates per 100,000 inhabitants in Santa Catarina and Joinville were 0.59 and 0.51, that is, below the national mortality rate, which was 0.63 ^30^.

This study had some limitations. It was not possible to measure the timing of the patient’s creatinine elevation to assess whether the interval to dialysis initiation, especially before ICU admission, may have impacted the outcome. Only one public hospital participated in the study, which may have limited the generalizability of the findings. Other public hospitals in the city were not referral centers for COVID-19 cases. The public hospital involved was a general hospital that served the entire macro-region of Joinville and had a healthcare structure similar to other large public hospitals. It was not possible to explore potential causes of mortality related to healthcare characteristics of each hospital through health indicators. Due to the retrospective nature of the study, information bias affecting the considered variables could not be excluded. All hospitals had systematic data collection routines through standardized patient history forms. This was the first study to compare survival and the impact of hospital type on the mortality of people with COVID-19 and dialysis-requiring AKI.

AKI was associated with a significant increase in the mortality risk among people with COVID-19. Low 90-day survival rates were evident among these people when treated in the public system in crude analyses. The risk of mortality at any time during the follow-up period was not higher in the public hospital when adjusted for the patient severity at ICU admission. These findings reinforced that measures adopted in Santa Catarina, may have minimized the intrinsic effects of the public healthcare system on critically ill people. The results of this study emphasize the need to a better understanding of care provided at other points in the public health network that may impact final outcomes for those requiring tertiary services.

## Data Availability

Not available.
