# Supplementary material for: Survival of critically ill people with COVID-19 and acute kidney injury undergoing hemodialysis in public and private hospitals in Joinville: a cohort study, 2020-2021
Source: Epidemiol Serv Saude. 2025 Apr 7;34:e20240025. doi: 10.1590/S2237-96222025v34e20240025.en (PMC11978279; doi:10.1590/S2237-96222025v34e20240025.en)
Supplement: Translation

# Translation: Sobrevida de pacientes críticos com covid-19 e injúria renal aguda em hemodiálise entre hospital público e privado de Joinville: estudo de coorte, 2020-2021

**Authors:** Elviani Basso Moura, Bruna de Albuquerque Catelano, Fernanda Perito de Aguiar, Helbert do Nascimento Lima, Paulo Henrique Condeixa de França

**Affiliations:** 1 Universidade da Região de Joinville, Programa de Pós-Graduação em Saúde e Meio Ambiente, Joinville, SC, Brasil

**Keywords:** Covid-19, Injúria Renal Aguda, Sistema Único de Saúde, Saúde Suplementar, Taxa de Sobrevida

## Abstract

**Objetivo:**

Comparar a sobrevida em 90 dias de pacientes críticos com covid-19 e injúria renal aguda em unidades de terapia intensiva (UTI) de hospitais público e privados.

**Métodos:**

Trata-se de coorte histórica de pacientes críticos em Joinville com Covid-19 e injúria renal aguda em hemodiálise.

**Resultados:**

A sobrevida em 90 dias na UTI pública foi 15,7% (IC95% 8,4; 25,1), e na privada, 37,7% (IC95% 24,9; 50,5). Na análise multivariada ajustada para variáveis sociodemográficas (hazard ratio (HR) 2,01; IC95% 1,31; 3,08) e comorbidades (HR 2,09; IC95% 1,30; 3,37), a internação em UTI pública associou-se ao maior risco de óbito. Após inclusão da gravidade, a internação em UTI pública não mostrou relação com risco aumentado de óbito comparado à UTI privada (HR 0,79; IC95% 0,45; 1,42).

**Conclusão:**

Medidas adotadas na pandemia de covid-19 podem ter reduzido desigualdades entre os sistemas de saúde em pacientes críticos com injuria renal aguda.

**Aspectos éticos**

A presente pesquisa respeitou os princípios éticos, obtendo os seguintes dados de aprovação:

Comitê de ética em pesquisa: Universidade da Região de Joinville

Número do parecer: 5.265.001

Data de aprovação: 25/2/2022

Certificado de apresentação de apreciação ética: 52712421.4.0000.5366

Termo de consentimento: Dispensado

## Introdução

A incidência de injúria renal aguda (IRA) é alta em pacientes com covid-19 tratados em unidade de terapia intensiva (UTI). A IRA foi descrita em 31,5% dos pacientes hospitalizados com covid-19, podendo alcançar 77,3% dos pacientes críticos 9 ^1^ ^,^ ^2^. O comprometimento renal implica aumento da morbimortalidade, maior tempo de internação e necessidade de diálise nos pacientes hospitalizados por covid-19 ^3^ ^,^ ^4^. A chance de pacientes com covid-19 e IRA morrerem é maior do que em indivíduos com covid-19 sem IRA ^5^.

Em um estudo retrospectivo com pacientes com covid-19 e IRA em UTI chegou-se a 75% dos casos, com metade dos pacientes necessitando de diálise e com mortalidade em 60 dias de até 67% ^6^. Fatores como idade avançada, sexo masculino, presença de diabetes, hipertensão, doença cardiovascular e insuficiência respiratória com necessidade de ventilação mecânica associaram-se à maior incidência de IRA em pacientes com covid-19 grave ^1^ ^,^ ^5^ ^,^ ^7^.

O enfrentamento da pandemia de covid-19 no Brasil foi marcado por desigualdades assistenciais em saúde e de acesso aos leitos hospitalares ^8^. Casos graves da doença causaram os maiores impactos sobre os sistemas de saúde, tanto público como privado, com o rápido aumento na demanda por internações hospitalares, especialmente em UTI ^9^. Grandes esforços dos gestores de saúde foram exigidos para o enfrentamento da covid-19 ^11^, visto que 80% das pessoas dependiam exclusivamente do Sistema Único de Saúde (SUS) no Brasil ^10^. 

A expansão do SUS tem sido um constante desafio para o rápido atendimento às necessidades de saúde da população brasileira, com o aumento dramático da cobertura dos serviços de saúde em apenas três décadas ^12^. São constatadas diferenças significativas de oportunidade de acesso e disposição de leitos de UTI no SUS (1,78 leito total e 0,25 leito de UTI para cada 1 mil habitantes), persistindo algumas iniquidades sanitárias ^13^. O SUS é o principal responsável por prover acesso da população às ações e aos serviços de saúde. O sistema também atende usuários de planos de saúde privados quando estes necessitam de atenção de alta complexidade não coberta integralmente pelo plano ^10^. 

Dados de mortalidade de pacientes críticos com covid-19 e IRA apontaram fragilidade do SUS, sendo de 65,4% versus 23,8% no serviço privado ^14^. Pouco se sabe sobre o impacto da IRA associada à covid-19 entre pacientes críticos atendidos pelo SUS quando comparado com aqueles atendidos pelo sistema privado de saúde.

Desafios emergiram na gestão dos serviços de saúde diante da eclosão da pandemia de covid-19, o que demandou a reorganização dos hospitais e da assistência prestada aos pacientes graves. Este estudo teve como objetivo comparar a sobrevida em 90 dias de pacientes críticos com covid-19 e IRA dependentes de hemodiálise atendidos pelo sistema de saúde público e privado em Joinville, a terceira cidade mais populosa do Sul do Brasil. Verificou-se se o tipo de atendimento, público ou privado, impactou a mortalidade nesses pacientes.

## Métodos

### Delineamento 

Trata-se de coorte retrospectiva com base em prontuário eletrônico. Foram incluídos todos os pacientes adultos internados em UTI de três hospitais de Joinville, Santa Catarina, com diagnóstico de covid-19 e que necessitavam de realizar hemodiálise aguda devido à IRA, entre março de 2020 e fevereiro de 2021. A infecção por covid-19 foi confirmada pelo exame molecular ou pelo teste rápido de antígeno. 

### Participantes

Três hospitais foram envolvidos no estudo. Dois eram privados (Hospital Dona Helena e Centro Hospitalar Unimed) e um era exclusivamente público (Hospital Municipal São José). Todas as UTI possuíam equipe assistencial multiprofissional, formada por médicos intensivistas, nutricionistas, enfermeiros e fisioterapeutas. Esses hospitais estavam vinculados a programas de residência médica. Na época do estudo, cada um deles ampliou sua estrutura de leitos em UTI para atender ao aumento de demanda de casos de covid-19. O Hospital Municipal São José contava com 40 leitos de UTI; o Hospital Dona Helena, com 18; e o Centro Hospitalar Unimed, com 20 leitos.

### Variáveis

Com base em informações contidas nos prontuários médicos, foram coletados dados demográficos (sexo e idade) e comorbidades atuais e prévias, como hipertensão, Diabetes, doença respiratória (doença pulmonar obstrutiva crônica ou asma), doença cerebrocardiovascular (acidente vascular cerebral, infarto agudo do miocárdio, revascularização do miocárdio ou angioplastia coronariana) e câncer. Coletaram-se dados referentes ao escore Simplified Acute Physiology Score III (SAPS-3), relativo à gravidade e à predição de mortalidade em UTI, além de necessidade e tempo de suporte ventilatório, presença de injúria renal aguda com necessidade de hemodiálise, tempo de internação na UTI e óbito durante a internação. 

A IRA foi definida por nefrologista de cada um dos hospitais envolvidos, e a indicação de hemodiálise foi decidida com base em parâmetros clínicos (hipervolemia) e laboratoriais (acidose metabólica, azotemia ou hipercalemia). Todos os pacientes com indicação de diálise foram submetidos a método intermitente de hemodiálise, com banho padrão de bicarbonato e por meio de acesso de curta permanência.

### Métodos estatísticos

As variáveis categóricas foram apresentadas por sua frequência e porcentagem. As variáveis quantitativas foram apresentadas por sua média e desvio padrão ou mediana e variação interquartil, conforme distribuição. Utilizou-se o teste do qui-quadrado ou teste exato de Fisher para comparar as variáveis categóricas. O teste Mann Whitney, após verificação de distribuição não normal pelo teste Kolmogorov-Smirnov, foi utilizado para comparar as médias das variáveis quantitativas segundo a variável de exposição principal, tipo de hospital (público ou privado), bem como entre os que sobreviveram ou não durante 90 dias de internação. O tempo de seguimento se deu da entrada do paciente na UTI até a ocorrência do óbito intra-hospitalar em até 90 dias. Pacientes sobreviventes que tiveram alta hospitalar antes de 90 dias foram censurados após o momento da alta. 

A análise da curva de sobrevida em relação ao tipo de hospital foi apresentada pelo método de Kaplan-Meier, e a diferença avaliada, pelo teste Log-Rank. Calculou-se hazard ratio (HR) quanto ao tipo de hospital de forma bruta e ajustada pelas principais variáveis de confundimento de forma bivariada por meio de regressão de Cox. Considerou-se variáveis de confundimento aquelas sabidamente associadas com a exposição principal ou que representavam fator de risco para o desfecho analisado. Todas as variáveis que modificaram o efeito entre a exposição principal (tipo de hospital) e o desfecho (óbito em 90 dias), em 5% ou mais na análise bivariada, foram incluídos no modelo multivariado. Para uma melhor visualização da mudança do efeito entre a variável de interesse principal com relação ao desfecho, as variáveis consideradas no modelo multivariado foram incluídas em blocos, sendo primeiramente as sociodemográficas, seguidas por comorbidades e complicações/gravidade do paciente em UTI. Todos os cálculos foram realizados no programa Stata/IC 15.1.

## Resultados

Durante o período considerado no estudo, 123 (22,9%) dos 538 pacientes internados em UTI nos hospitais públicos e privados com covid-19 apresentaram IRA com necessidade de hemodiálise aguda. Setenta pacientes (56,9%) foram internados em hospital público, e 53 (49,1%), em hospitais privados.

A mediana de idade da amostra total analisada foi 63 anos, sendo 79 (64,2%) homens. As comorbidades mais presentes foram hipertensão (n=72; 58,5%) e diabetes (n=44; 35,8%). O uso de ventilação mecânica foi necessário em 118 (95,9%) casos, a mediana do SAPS-3 foi 67 (variação interquartil 52/76) e 92 (74,8%) pacientes foram a óbito em 90 dias. Comparado aos pacientes em hospital privado, os pacientes de hospital público tiveram menor prevalência de doença pulmonar obstrutiva crônica ou asma (5,7% vs. 22,6%; p-valor 0,007), maior necessidade de ventilação mecânica (100% vs. 90.6%; p-valor 0,009), menor mediana do tempo em UTI (17,5 vs. 23 dias; p-valor 0,022), menor mediana do tempo de ventilação mecânica (17 vs. 21, 5 dias; p-valor 0,031), maior mediana da gravidade (73,5 vs. 52; p-valor<0,001) e maior taxa de mortalidade (84,3% vs. 62,3%; p-valor 0,005) (Tabela 1). Não houve diferença quanto a idade entre os pacientes de hospital público (mediana de 65 anos, intervalo interquartil 54-73) e privado (mediana 60 anos; intervalo interquatil 47-73; p-valor 0,199). O tempo de UTI no hospital privado (21 dias, intervalo interquartil 12-38) foi maior comparado aos pacientes de hospital público (17,5 dias, intervalo interquartil 10-29; p-valor 0,022). Também o tempo de ventilação mecânica foi maior nos pacientes de hospital privado (21,5 dias, intervalo interquartil 14-45) comparado aqueles de hospital público (17 dias, intervalo interquartil 10-24; p-valor 0,031). Já, a gravidade foi maior nos pacientes de hospital público (73,5, intervalo interquartil 64-78) comparado aos que estavam em hospital privado (52, intervalo interquartil 49-64; p-valor<0,001).

**Tabela 1:** Características dos pacientes com covid-19 e injúria renal aguda com necessidade de hemodiálise pelo tipo de hospital. Joinville, 2020-2021 (n=123)

Variáveis	Privado (%)	Público (%)	p-valor	Total (%)
Sexo masculino	38 (71,7)	41 (58,6)	0,133	79 (64,2)
Hipertensão	35 (66,0)	37 (52,9)	0,142	72 (58,5)
Diabetes	19 (35,8)	25 (35,7)	0,988	44 (35,8)
Doença respiratória	12 (22,6)	4 (5,7)	0,007	16 (13,0)
Tabagismo	8 (15,1)	11 (15,7)	0,925	19 (15,4)
Câncer	8 (15,1)	4 (5,7)	0,077	12 (9,8)
Doença cerebrocardiovascular	6 (11,3)	3 (4,3)	0,129	9 (7,3)
Uso de drogas vasoativas	32 (60,4)	48 (68,6)	0,345	80 (65,0)
Ventilação mecânica	48 (90,6)	70 (100,0)	0,009	118 (95,9)
Óbito	33 (62,3)	59 (84,3)	0,005	92 (74,8)

Comparado aos sobreviventes, os pacientes que foram a óbito tinham maior média de idade (66,5 vs. 60,1 anos; p-valor 0,009), maior frequência no uso de drogas vasodilatadoras (76,1% vs. 32,3%; p-valor <0,001), maior necessidade de ventilação mecânica (100% vs. 83,9%; p-valor<0,001) e menor mediana do tempo de ventilação mecânica (17 vs. 30,5 dias; p-valor 0,006) e de UTI (17 vs. 42 dias; p-valor 0,001). A mediana do escore SAPS-3 foi mais elevada entre os pacientes que vieram a óbito (70 vs. 58; p-valor 0,005; Tabela 2). Houve diferença quanto a idade entre os que sobreviveram (mediana de 55 anos, intervalo interquartil 44-66) e aqueles que vieram a óbito (mediana de 70 anos; intervalo interquatil 60-77; p-valor 0,009). O tempo de UTI entre os que sobreviveram também foi maior (42 dias, intervalo interquartil 22-89) comparado àqueles que morreram (17 dias, intervalo interquartil 11-28; p-valor 0,001). Também o tempo de ventilação mecânica foi maior entre os que sobreviveram (30,5 dias, intervalo interquartil 18-52) comparado aos que morreram (17 dias, intervalo interquartil 10-24; p-valor 0,006). Já, a gravidade foi menor entre os que sobreviveram (58, intervalo interquartil 49-67) comparado aos que vieram a óbito (70, intervalo interquartil 54-7-; p-valor 0,005).

**Tabela 2:** Características gerais dos pacientes com covid-19 e injúria renal aguda com necessidade de hemodiálise com relação à sobrevida em até 90 dias. Joinville, 2020-2021 (n=123)

Variáveis	Sobrevivência (%)	Óbito (%)	p-valor
Sexo masculino	16 (51,6)	63 (68,5)	0,090
Hipertensão	22 (71,0)	50 (54,3)	0,104
Diabetes	11 (35,5)	33 (35,9)	0,969
Doença respiratória	4 (12,9)	12 (13,0)	0,627
Tabagismo	2 (6,4)	17 (18,5)	0,089
Câncer	2 (6,4)	10 (10,9)	0,374
Doença cerebrocardiovascular	3 (9,7)	6 (6,5)	0,405
Uso de drogas vasoativas	10 (32,3)	70 (76,1)	<0,001
Ventilação mecânica	26 (83,9)	92 (100,0)	<0,001
Hospital privado	20 (64,5)	33 (35,9)	0,005
Hospital público	11 (35,5)	59 (64,1)	0,005

Houve diferença no tempo de sobrevida entre os pacientes com covid-19 e IRA com necessidade de hemodiálise quanto ao tipo de hospital quando analisado pelo método Kaplan Meier (p-valor 0,002; Figura 1). A sobrevida em 90 dias dos pacientes internados em hospital público foi 15,7% (IC95% 8,4; 25,1). A sobrevida em 90 dias dos pacientes que internaram em hospital privado foi 37,7% (IC95% 24,9; 50,5). 

Pacientes internados em hospital público apresentaram 90% maiores chances de óbito comparado àqueles em hospital privado (HR 1,93; IC95% 1,26; 2,97). Após ajuste diante das demais variáveis, o efeito referente ao tipo de hospital foi modificado em ≥5% quando ajustado para câncer (HR 2,06; IC95% 1,32; 3,20), doença pulmonar obstrutiva crônica ou asma (HR 2,01; IC95% 1,29; 3,05), sexo (HR 1,98; IC95% 1,29; 3,05) e idade (HR 1,96; IC95% 1,28; 3,02; Tabela 3).

**Tabela 3:** Hazard ratio (HR) e intervalo de confiança (IC95%) bruta e ajustada entre tipo de hospital e óbito em 90 dias de pacientes com covid-19 e injúria renal aguda com necessidade de hemodiálise. Joinville, 2020-2021 (n=123)

Variáveis	HR bruta (IC95%)	p-valor	HR ajustada (IC95%)	p-valor
**Hospital**				
Privado	1,00			
Público	1,93 (1,26; 2,97)	0,003		
**Sexo**				
Homem			1,00	
Mulher			1,98 (1,29; 3,05)	0,002
**Hipertensão**				
Não			1,00	
Sim			1,85 (1,19; 2,86)	0,006
**Diabetes**				
Não			1,00	
Sim			1,93 (1,26; 2,97)	0,003
**Doença respiratória**				
Não			1,00	
Sim			2,01 (1,29; 3,14)	0,002
**Doença cerebrocardiovascular**				
Não			1,00	
Sim			1,92 (1,24; 2,96)	0,003
**Câncer**				
Não				
Sim			2,06 (1,32; 3,20)	0,001
**Uso de drogas vasoativas**				
Não			1,00	
Sim			1,68 (1,09; 2,59)	0,019
**Tabagismo**				
Não			1,00	
Sim			1,91 (1,24; 2,94)	0,003
**Idade (anos de aumento)**			1,96 (1,28; 3,02)	0,002
**Tempo de ventilação mecânica (dias/unidade de aumento)**			1,16 (0,75; 1,79)	0,501
**Gravidade (unidade de aumento)**			1,35 (0,79; 2,30)	0,276

Nota: ^a^Anemia, dor crônica, gota, lúpus, miomatose uterina e paralisia infantil.

**Figura 1:** Sobrevida em 90 dias de pacientes com covid-19 e injúria renal aguda com necessidade de hemodiálise pelo tipo de hospital. Joinville, 2020-2021

A internação em hospital público se manteve associada ao maior risco de óbito apenas quando ajustado pelas variáveis sociodemográficas (HR 2,01; IC95% 1,31; 3,08) e quando adicionado no modelo a presença de comorbidades (HR 2,09; IC95% 1,30; 3,37; Tabela 4). Quando ajustado para gravidade, a internação de pacientes críticos com covid-19 e IRA com necessidade dialítica em hospital público não apresentou relação ao maior risco de mortalidade (HR 0,79; IC95% 0,45; 1,42).

**Tabela 4:** Hazard ratio (HR) e intervalo de confiança (IC95%) de modelo multivariado entre tipo de hospital e óbito em 90 dias de pacientes com covid-19 e injúria renal aguda com necessidade de hemodiálise. Joinville, 2020-2021 (n=123)

Variáveis	Modelo 1			Modelo 2			Modelo final
	HR (IC95%)	p-valor	HR (IC95%)		p-valor	HR (IC95%)	p-valor
**Tipo de hospital (público/privado)**	2,01 (1,31; 3,08)	0,001	2,09 (1,30; 3,37)		0,003	0,79 (0,45; 1,42)	0,440
**idade (ano de aumento)**	1,02 (1,01; 1,04)	0,006	1,02 (1,00; 1,04)		0,015	1,01 (0,99; 1,03)	0,152
**Sexo**							
Masculino	1,00		1,00			1,00	
Feminino	0,76 (0,48; 1,18)	0,219	0,78 (0,49; 1,24)		0,297	0,90 (0,56; 1,44)	0,654
**Diabetes**							
Sim			1,00			1,00	
Não			0,99 (0,64; 1,53)		0,949	1,01 (0,64; 1,59)	0,976
**Doença respiratória**							
sim			1,00			1,00	
não			1,19 (0,6; 2,30)		0,614	1,31 (0,64; 2,69)	0,460
**Doença cerebrocardiovascular**							
sim			1,00			1,00	
não			0,78 (0,33; 1,84)		0,567	0,85 (0,35; 2,02)	0,706
**Câncer**							
sim			1,00			1,00	
não			1,25 (0,58; 2,67)		0,569	1,64 (0,74; 3,63)	0,223
**Uso de droga vasoativa**							
sim						1,00	
não						1,08 (0,64; 1,84)	0,768
**Tempo de ventilação mecânica (dias/unidade de aumento)**						0,96 (0,94; 0,97)	<0,001
**Gravidade (unidade de aumento)**						1,03 (1,00; 1,05)	0,013

## Discussão

Evidenciou-se a marcante menor sobrevida em 90 dias de pacientes críticos com covid-19 e IRA com necessidade dialítica internados em hospital público. Após ajuste para a gravidade de tais pacientes no momento da admissão da UTI, o tipo de hospital não impactou o maior risco de óbito em 90 dias.

A presença de IRA com necessidade dialítica como fator complicador de pacientes críticos internados por covid-19 demonstrou decréscimo ao longo da pandemia, variando entre 27% no primeiro ano para 14% no período final da pandemia ^15^. Neste estudo, a prevalência geral de IRA com necessidade de diálise foi 22,9% dos pacientes que necessitaram de UTI. A presença de IRA com necessidade dialítica em pacientes críticos com covid-19 no hospital público foi 61,5% ^2^, enquanto, em um hospital privado, foi 17% ^14^. Tal variação pôde ser parcialmente explicada pelos critérios definidos para indicação de diálise e perfil de gravidade dos pacientes admitidos na UTI. Entre as características associadas com a ocorrência de IRA em pacientes com covid-19, destacam-se o aumento da idade, o sexo masculino, a presença de comorbidades, como hipertensão, diabetes e doença cardiovascular, a maior necessidade de ventilação mecânica e a maior gravidade ^16^ ^-^ ^18^. Esses fatores foram encontrados neste estudo entre os pacientes com IRA dialítica. 

O uso da ventilação mecânica em pacientes críticos com covid-19 foi apontado como importante fator de risco para IRA ^14^ ^,^ ^19^. Em uma coorte multicêntrica em hospitais públicos no Brasil, o uso dessa ventilação foi um dos fatores preditores para o maior risco de mortalidade nos pacientes com covid-19 ^20^. Observou-se que os pacientes internados no hospital público necessitaram de maior frequência de uso de ventilação mecânica com menor tempo de internação do que no privado. Esse menor tempo de internação associou-se ao maior número de óbitos no serviço público. 

A chance de sobrevida em 90 dias foi mais do que o dobro superior para os pacientes atendidos em hospitais privados, quando analisada de forma bruta a sobrevida entre os pacientes críticos com covid-19 e IRA com necessidade de diálise neste estudo. Entre os aspectos que poderiam estar associados à menor sobrevida entre os pacientes dependentes do SUS, destacou-se o uso de drogas vasodilatadoras e da ventilação mecânica. Outras variáveis que poderiam implicar essa menor sobrevida no SUS, mas não avaliadas neste estudo, seriam as desigualdades sociais de base ^10^ e os aspectos técnicos relacionados à assistência ^17^ ^,^ ^21^. O tempo de acesso e o tipo de cuidado antes da internação na UTI poderiam ter impacto nessa diferença encontrada na sobrevida. As características individuais e as condições de saúde e de acesso aos serviços de saúde puderam determinar a sobrevida dos indivíduos infectados pela covid-19 ^17^ ^,^ ^18^ ^,^ ^22^ ^,^ ^23^.

Comparou-se o risco de morte em qualquer momento durante o período de observação, ajustado pelas variáveis sociodemográficas e comorbidades. Notou-se que o hospital público se manteve associado ao maior risco de óbito. O risco de morte por covid-19 foi atribuído à alta probabilidade de superlotação nos serviços públicos, ao acesso desigual à saúde, às piores condições de vida e à maior prevalência de comorbidades ^24^. Destacou-se que ser atendido em hospital público no Brasil foi fator independente para mortalidade ^25^. 

O risco de óbito para pacientes com covid-19 e IRA com necessidade dialítica não se mostrou diferente segundo o tipo de hospital ajustado para variáveis sociodemográficas e comorbidades, quando o tipo de hospital também foi ajustado pela gravidade. As variáveis utilizadas para comprovar gravidade foram ventilação mecânica e escore de mortalidade SAPS-3. A ventilação mecânica foi associada ao risco de mortalidade por covid-19 ^26^, e o escore SAPS-3, à maior mortalidade hospitalar em pacientes com covid-19 internados em UTI ^27^. Percebeu-se que a mortalidade ajustada por outros fatores, e considerando a gravidade na chegada do paciente à UTI, foi semelhante nos dois tipos de hospitais neste estudo. 

O tipo de plano de saúde não influenciou o acesso à UTI dos pacientes com covid-19 e insuficiência renal crônica já em programa permanente de hemodiálise ^12^. A mortalidade foi maior entre os que necessitaram de UTI pública comparado à UTI privada ^12^. As maiores gravidade na admissão à UTI e necessidade de ventilação mecânica, entre os pacientes que necessitaram de UTI pública neste estudo, podem ter corroborado para que o risco de mortalidade não fosse diferente daqueles que necessitaram de UTI privada. Não se permitiu afirmar que a pior situação do paciente que era admitido à UTI pública não fora reflexo das dificuldades do atendimento inicial prestado nos demais pontos da rede de atenção pública.

A ampliação emergencialmente de estruturas assistenciais e de leitos de UTI pôde ter reduzido os impactos da covid-19 para o sistema público de saúde. Acreditou-se que fatores organizacionais, incluindo a implementação de protocolo e a otimização do uso de recursos nas UTIs, foram medidas potenciais para melhorar os resultados dos pacientes, com redução de mortalidade hospitalar ^28^. Os leitos de UTI adulto no SUS representaram a maioria dos leitos de UTI adulto no país (67%) destinados à covid-19 no período do estudo. Destacou-se Santa Catarina, onde 94% dos leitos de UTI eram do SUS ^13^. 

As medidas adotadas pelo sistema público de saúde em Joinville foram destaques, como a criação de grupo técnico de enfrentamento, a ampliação de UTIs e a contratação de mais profissionais de saúde. Ocorreu também a compra de equipamentos para atendimento de casos graves ^29^, que podem ter contribuído para minimizar os desfechos negativos aos pacientes graves com covid-19 internados na UTI. Como mostraram os dados gerais da covid-19 no Brasil, as taxas de mortalidade por 100 mil habitantes de Santa Catarina e de Joinville foram 0,59 e 0,51, ou seja, abaixo da taxa de mortalidade nacional, que foi 0,63 ^30^.

Este estudo teve algumas limitações. Não foi possível mensurar em qual momento da internação o paciente iniciou com aumento da creatinina para avaliar se o tempo para início da diálise, principalmente antes do acesso à UTI, pode ter impactado o desfecho. Houve a participação de somente um hospital público na pesquisa, o que pode ter limitado a generalização destes achados. Os demais hospitais da rede pública na cidade não eram referência para casos de covid-19. O hospital público envolvido era um hospital geral que atendia a toda macrorregião de Joinville e possuía estrutura assistencial semelhante a outros hospitais públicos de grande porte. Não foi possível explorar potenciais causas de mortalidade que pudessem estar relacionadas às características assistenciais de cada hospital por meio de indicadores em saúde. Pela própria natureza retrospectiva do estudo, não se pôde afastar a existência de viés de informação com impacto nas variáveis consideradas. Todos os hospitais tinham rotina para coleta de dados por meio de anamnese sistematizada. Este estudo foi o primeiro a comparar a sobrevida e o impacto do tipo de hospital na mortalidade de pacientes com covid-19 e IRA com necessidade dialítica. 

A IRA associou-se ao aumento significativo no risco de mortalidade nos pacientes com covid-19. Evidenciou-se a baixa sobrevida em 90 dias desses pacientes ao serem assistidos pelo sistema público quando considerado de forma bruta. O risco de mortalidade em qualquer momento do período considerado de seguimento não se mostrou maior no hospital público quando ajustado para a gravidade do paciente à internação em UTI. Esses achados reforçaram que a adoção de medidas em Santa Catarina pode ter minimizado os efeitos intrínsecos à rede pública aos pacientes graves. Os resultados deste estudo enfatizam a necessidade de compreender melhor a assistência prestada em outros pontos de atenção da rede pública de saúde que possam impactar o desfecho final àqueles que necessitam do serviço terciário.

## Data Availability

Não disponível.
